# High-risk HPV infection-associated hypermethylated genes in oropharyngeal squamous cell carcinomas

**DOI:** 10.1186/s12885-022-10227-w

**Published:** 2022-11-07

**Authors:** Yoshikuni Inokawa, Masamichi Hayashi, Shahnaz Begum, Maartje G. Noordhuis, Daivd Sidransky, Joseph Califano, Wayne Koch, Mariana Brait, William H. Westra, Mohammad O. Hoque

**Affiliations:** 1 Department of Otolaryngology-Head and Neck Surgery, Johns Hopkins University School of Medicine, Baltimore, MD USA; 2 Department of Gastroenterological Surgery, Nagoya University Graduate School of Medicine, Nagoya, Aichi Japan; 3 Department of Pathology, Johns Hopkins University School of Medicine, Baltimore, MD USA; 4 Division of Otolaryngology-Head and Neck Surgery, Department of Surgery, University of California San Diego, CA San Diego, USA; 5 Department of Pathology, Molecular and Cell-Based Medicine, Icahn School of Medicine at Mount Sinai, New York, NY USA; 6 Department of Oncology, Johns Hopkins University School of Medicine, Baltimore, MD USA; 7 Department of Urology, Johns Hopkins University School of Medicine, Baltimore, MD USA; 8 Department of Otolaryngology and Head & Neck Surgery, Urology and Oncology, The Johns Hopkins University School of Medicine, 1550 Orleans Street, CRB II, 5M, Baltimore, MD 21231 USA

**Keywords:** HPV, Oropharyngeal SCC, Methylation, AGTR1

## Abstract

**Background:**

HPV-positive oropharyngeal squamous cell carcinomas (OPSCCs) are sensitive to chemo-radiation therapy and have favorable survival outcomes compared with HPV-negative cancers. These tumors are usually not related to tobacco and alcohol exposure. Therefore, diagnosing HPV-positive OPSCCs for the appropriate disease management is crucial, and no suitable markers are available for detecting early malignancies in HPV-infected tissues. In this study, we attempt to find HPV-specific epigenetic biomarkers for OPSCCs.

**Methods:**

A total of 127 surgical samples were analyzed for HPV positivity and promoter methylation of a panel of genes. HPV detection was performed by PCR detection of HPV E6 and E7 viral oncoproteins. In addition, promoter methylation of a total of 8 genes (*DAPK*, *FHIT*, *RASSF1A*, *TIMP3*, *AGTR1*, *CSGALNACT2*, *GULP1* and *VGF*) was analyzed by quantitative-methylation specific PCR (QMSP), and their associations with HPV positivity or RB/p16 expressions were evaluated.

**Results:**

*AGTR1* and *FHIT* were frequently methylated in HPV-positive OPSCC samples with a good area under the curve (AUC over 0.70). In addition, these genes' promoter methylation was significantly associated with p16 positive and RB negative cases, which were the characteristics of OPSCC cases with favorable survival outcomes. Either *AGTR1* or *FHIT* methylated cases were significantly associated with HPV-positive cancers with 92.0% sensitivity (*P* < 0.001). Also, they had significantly better overall survival (*P* = 0.047) than both unmethylated cases.

**Conclusions:**

A combination of *AGTR1* and *FHIT* methylation demonstrated a suitable detection marker of OPSCCs derived from the HPV-infected field, familiar with p16-positive and RB-negative phenotypes.

**Supplementary Information:**

The online version contains supplementary material available at 10.1186/s12885-022-10227-w.

## Introduction

Oral Human papillomavirus (HPV) infection is strongly associated with oropharyngeal squamous cell carcinoma (OPSCC) [1, 2]. About 40–80% of oropharyngeal cancers (OCs) are caused by HPV in the USA, whereas the prevalence of HPV infection-associated OCs varies from 20 to 90% in Europe [3]. The incidence of OPSCC typically occurs at tonsil and tongue base [3]. Carcinogenesis of HPV-positive OCs is characterized by TP53-degradation, retinoblastoma (RB) pathway-inactivation and p16-upregulation [3]. Briefly, HPV infection dysregulates the oncoprotein expressions of E6 and E7. E6 protein binds to P53 and promotes its degradation [4, 5]. E7 protein binds and inactivates pRB releasing E2F from the pRB-E2F complex, and E2F activates the cell cycle. Disruption of the pRB-E2F complex also releases p16 from its negative feedback control, resulting in p16 overexpression. Since pRB is degraded and E2F is unbound, p16 no longer has an inhibitory function on pRB and cannot inhibit the cell cycle. By contrast, the carcinogenesis process of HPV-negative OCs is different. Typically, tobacco-related OCs are characterized by TP53 mutation and down-regulation of p16. Thus, HPV-positive and HPV-negative OCs have obviously different kinds of carcinogenesis pathways.

Epigenetic alterations of critical cancer-associated genes are considered as a hallmark of cancer. Van Kempen PM et al. reported a systematic review of differential methylation profiles between HPV-positive and HPV-negative OPSCC [6] in 2014. They summarized differential methylated genes between HPV- positive and HPV-negative cases. Accumulated evidence suggests that promoter methylation more frequently occurs in HPV-positive tumors than HPV-negative tumors. However, no specific panel of methylation markers has yet been identified for potential clinical use.

The primary purpose of this study was to identify differentially methylated candidate genes between HPV-positive OPSCCs and others. They could be used as markers for non-invasive early molecular detection approaches (screening) using bodily fluids (such as saliva and blood) and non-invasive monitoring of recurrent disease. The secondary purpose was to find the correlation of methylation markers with representative HPV-related carcinogenesis pathway factors, p16 and RB.

## Materials and methods

### Clinical sample collection

A total of 94 patients undergoing surgical resection of primary untreated OPSCCs at The Johns Hopkins Hospital from 1997 to 2008 were included. Written informed consent was obtained from all subjects and their legal guardians recruited under this protocol before participation in the study. All samples were obtained as anonymized materials following the Declaration of Helsinki. They were classified into 50 HPV-positive and 44 HPV-negative cases using the PCR detection method focusing on HPV E6 and E7 regions of HPV type 16 following the previous paper from our lab [7]. In addition, we also used 33 non-neoplastic tonsil tissues from other non-cancer patients cohort as a control. Details of these samples are available in Table 1.

**Table 1:** Clinicopathological characteristics of study cohort

Factors	Control (*n* = 33)	OPSCC (*n* = 94)	*P* values	HPV-positive (*n* = 50)	HPV-negative (*n* = 44)	*P*-value
Age (> 60/ < 60)	1/32	37/57	** < 0.001**	16/34	21/23	0.142
Sex (Male/Female)	24/9	82/12	0.062	48/2	34/10	**0.011**
Race (White/Other)	26/6 (1)	74/20	1.000	42/8	32/12	0.213
Smoking (Yes/No)	6/22 (5)	71/14 (9)	** < 0.001**	33/11 (6)	38/3 (3)	**0.040**
Alcohol (Yes/No)	4/24 (5)	48/37 (9)	** < 0.001**	18/26 (6)	30/11 (3)	**0.004**
Stage (I-III/IV)	N/A	25/69	N/A	10/40	15/29	0.162
Grade (1–2/3)	N/A	75/19	N/A	39/11	36/8	0.798

(number): data not available in the patients’ database, Control: Non-neoplastic tonsil tissues, Smoking: Yes/Quit versus No, Alcohol Yes versus No/Socially drinking, *P* values were analyzed by Fisher’s exact test, two-tailed. Significant *P* values were bolded

*N/A* Not applicable

### DNA extraction and bisulfite modification

Hematoxylin and eosin-stained sections were histologically examined to evaluate the presence of tumor cells. Sample sections showing more than 70% of tumor cells were used for DNA extraction. Microdissected tissues were digested with 1% SDS and 50 μg/ml proteinase K (Boehringer Mannheim, Germany) at 48 °C overnight. Phenol and chloroform extraction and ethanol precipitation of DNA were performed as previously described [8]. Then, sodium bisulfite-mediated conversion of unmethylated cytosines in DNA was performed by EpiTect Bisulfite Kit (Qiagen, Venlo, Netherlands).

### Candidate genes

A total of 8 genes were selected for promoter methylation analysis. Among these eight genes, four genes are highly methylated in head and neck squamous cell carcinomas (HNSCCs) as others, and we reported previously [*DAPK* [9], *FHIT* [10], *RASSF1A* [11, 12] and *TIMP3* [13, 14]]. The remaining four novel methylation markers (*AGTR1* [15], *CSGALNACT2*, *GULP1* [16, 17] and *VGF* [18, 19]) were chosen from our recent high throughput “methylome” study based on their cancer relevance from microarray data analyses [16, 18, 20]. Chromosomal locus, proposed function and known association with cancer of these genes were summarized in Supplementary Table S1.

### Quantitative methylation-specific PCR (QMSP)

Bisulfite-modified genomic DNA samples served as templates for QMSP. Primers and probes were listed in Supplementary Table S2. Serial dilutions (90–0.009 ng) of CpG methyltransferase (New England BioLabs, Ipswich, MA) were used to construct a calibration curve for each plate. Also, negative controls (human leukocyte genomic DNA from a healthy donor) and multiple water blanks were placed [21]. Amplification reactions were carried out in triplicate in a final volume of 20 μl containing 3 μl bisulfite-modified DNA, 600 nmol/l forward and reverse primers, 200 nmol/l probes, 0.6 unit Platinum *Taq* D (Invitrogen), dATP, dCTP, dGTP, and dTTP in a concentration of 200 μmol/l each and 6.7 mmol/l MgCl_2_. Amplification reactions were carried out in 384-well plates in a 7900HT Fast Real-Time PCR System (Life Technologies, Carlsbad, CA) and were analyzed by the Sequence Detector System software (SDS 2.3; Life technologies). The relative level of methylated DNA for each gene in each sample was determined as a ratio of the QMSP value of the amplified gene to *ACTB*, multiplied by 1000 for easy tabulation, following the protocol we previously published [22].

### Immunohistochemical staining (IHC)

IHC for RB was performed using the G3-245 mouse monoclonal antibody (BD Biosciences, San Jose, CA) at a dilution of 1:2000 after 20 min of steaming in 10 mM citrate buffer. Labeling was visualized using the DAKO LSAB kit (DAKO, Carpinteria, CA). The percentage of neoplastic cell nuclei labeling in each case was assessed. Slides were considered RB negative when no neoplastic cell nucleus was stained. Detailed procedures and standardized IHC photos for RB staining were shown in a previous paper [23]

IHC for p16 was performed using the monoclonal antibody 16PO4 at a dilution of 1:100 (Cell Marque Inc, Hot Springs, AR). Positive p16 labeling was defined as the presence of nuclear and cytoplasmic reactivity. The percentage of cells showing nuclear and cytoplasmic labeling was recorded. The detailed procedure and standardized IHC photos for p16 staining were shown in the previous paper [24].

For both antibodies, negative controls were performed by omitting the primary antibody incubation step. The staining intensity was evaluated by a senior pathologist and scored as 1 (Strong over 50%), 2 (Stained in 20–50%), 3 (Weak), or 4 (Negative). In addition, we defined p16 or RB expression level as follows: Positive (scores 1 and 2) and Negative (scores 3 and 4) because sample distributions were dramatically changed between HPV positive and negative samples by this threshold (Supplementary Table S4).

### Statistics

Continuous variables were analyzed by the Mann–Whitney U test, and categorical variables were analyzed by Fisher’s exact test, two-tailed. Disease-specific survival was defined as the time from surgery to the date of cancer death. Those who remained alive were censored at the last date the subject was known to be alive. The Association of gene methylation with OS was evaluated using the Cox proportional hazards model with hazard ratios and 95% confidence intervals estimated for multivariable analysis. The individual gene’s methylation level cut-off value was determined by maximizing the sensitivity and specificity using Receiver Operating Characteristics (ROC) curve analysis. All statistical analyses were performed using JMP 16 software (SAS Institute, Cary, U.S.A.). The level of statistical significance was set at *P* < 0.05.

## Results

### Characteristics of the study cohort

A total of 127 surgical samples were included in this study. The incidence of HPV-positive and HPV-negative cases is significantly different considering gender, smoking history, and alcohol consumption (Table 1). These findings seem typical of HPV-positive OPSCCs, which are known to be seen in young men without tobacco or alcohol use compared to HPV-negative OPSCCs.

### HPV-positive OPSCC-specific methylated genes

**Fig. 1:**
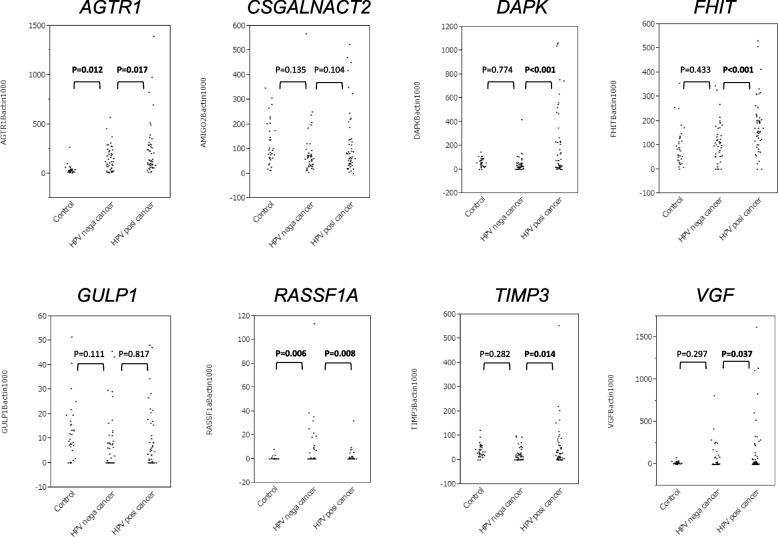
Scattered plots of QMSP values of tested genes in control samples (*n* = 33), HPV negative OPSCCs (*n* = 44) and HPV positive OPSCCs (*n* = 50)

We established an optimal cut-off value for each of the eight genes using 50 HPV-positive OPSCC cases and 77 others, including 44 HPV-negative OPSCCs and 33 controls by ROC curve analysis (Fig. 1, Supplementary Figure S1). All HPV-positive OPSCC cases and others were dichotomized into high and low methylated cases based on the cut-off value. The cut-off value and AUC for each of the eight genes are listed in Table 2. All genes, except for *CSGALNACT2*, *GULP1* and *RASSF1A,* showed significantly higher methylation frequencies in HPV-positive OPSCC cases than others. Mainly, *AGTR1* (92% and 48%, *P* < 0.001) and *FHIT* (68% and 25%, *P* < 0.001) were frequently methylated in HPV-positive OPSCCs compared to others with fair AUC values (> 0.700). Interestingly, *GULP1* and *RASSF1A* promoter methylation frequency was significantly higher in HPV-negative cases than in HPV-positive cases.

**Table 2:** HPV positive OPSCC specific methylation markers

Gene	AUC	Cut-off	HPV positive OPSCCs (*n* = 50) Methylated status (High / Low)	Others (*n* = 77) Methylated status (High / Low)	***P* value**
*AGTR1*	0.735	44.4	92% (46/4)	48% (37/40)	** < 0.001**
*CSGALNACT2*	0.520	80.3	52% (26/24)	39% (30/47)	0.200
*DAPK*	0.636	118.5	44% (22/28)	5% (4/73)	** < 0.001**
*FHIT*	0.735	136.2	68% (34/16)	25% (19/58)	** < 0.001**
*GULP1*	0.558	5.4	42% (21/29)	58% (45/32)	0.101
*RASSF1A*	0.511	9.4	4% (2/48)	13% (10/67)	0.124
*TIMP3*	0.546	71.0	22% (11/39)	8% (6/71)	**0.032**
*VGF*	0.603	12.7	60% (30/20)	36% (28/49)	**0.011**

*P* values were analyzed by Fisher's exact test, two-tailed. Significant *P* values were bolded. Methylated status (%): percentage of highly methylated cases

The association of promoter methylation of candidate genes with other clinicopathological parameters is shown in Supplementary Table S3. *AGTR1* and *RASSF1A* methylation were associated with advanced age, while *CSGALNACT2*, *FHIT* and *GULP1* were associated with race. No drinking history affects *DAPK* methylation, and *VGF* methylation influences tumor grades 1–2.

### Univariate analysis of disease-specific survival of 94 OPSCCs

The Cox proportional hazards model was used for univariate analysis to find prognostic factors of disease-specific survival of 94 OPSCCs. Age, race, smoking, alcohol consumption, HPV negative, p16 negative and RB positive were significant factors of disease-specific survival (Table 3). Some of the Kaplan–Meier curves are shown in Supplementary Figure S2. These factors were significantly correlated with HPV-negative OPSCCs but not with HPV-positive OPSCCs. None of the methylation markers was a significant prognostic factor, neither for HPV-positive nor HPV-negative OPSCCs. In the multivariable analysis, Age over 60 years old and positive RB were the significant prognostic factor of disease-specific survival.

**Table 3:** Univariate analysis of disease-specific survival of 94 OPSCCs (Cox proportional hazards model)

Factors	Univariate analysis	Multivariable analysis
Age (> 60/ < 60)	** > 60, HR = 2.69, *P* = 0.004, 95%CI:1.39–5.33**	** > 60, HR = 2.45, *P* = 0.031, 95%CI:1.08–5.53**
Sex (Female/Male)	Female, HR = 1.76, *P* = 0.205, 95%CI:0.73–4.25	
Race (White/Other)	**Other, HR = 2.13, *P* = 0.047, 95%CI:1.01–4.48**	
Smoking (Yes/No)	**Yes, HR = 3.48, *P* < 0.001, 95%CI:1.70–7.16**	
Alcohol (Yes/No)	**Yes, HR = 3.52, *P* < 0.001, 95%CI:1.67–8.12**	
Stage (I-III/IV)	Stage IV, HR = 1.56, *P* = 0.291, 95%CI:0.68–3.56	
Grade (1–2/3)	Grade3, HR = 1.18, *P* = 0.700, 95%CI:0.51–2.70	
HPV (Positive/Negative)	**Negative, HR = 3.45, *P* < 0.001, 95%CI:1.76–7.05**	
p16 (Positive/Negative)	**Negative, HR = 2.65, *P* = 0.005, 95%CI:1.35–5.42**	
RB (Positive/Negative)	**Positive, HR = 3.27, *P* = 0.004, 95%CI:1.46–7.79**	**Positive, HR = 3.38, *P* = 0.004, 95%CI:1.48–7.73**
*AGTR1* (High/Low)	Low, HR = 2.10, *P* = 0.067, 95%CI:0.95–4.65	
*CSGALNACT2* (High/Low)	Low, HR = 1.49, *P* = 0.254, 95%CI:0.75–2.96	
*DAPK* (High/Low)	Low, HR = 2.27, *P* = 0.054, 95%CI:0.99–5.20	
*FHIT* (High/Low)	Low, HR = 1.12, *P* = 0.736, 95%CI:0.58–2.18	
*GULP1* (High/Low)	High, HR = 1.18, *P* = 0.622, 95%CI:0.61–2.28	
*RASSF1A* (High/Low)	High, HR = 1.65, *P* = 0.267, 95%CI:0.68–3.97	
*TIMP3* (High/Low)	Low, HR = 1.61, *P* = 0.367, 95%CI:0.57–4.56	
*VGF* (High/Low)	Low, HR = 1.04, *P* = 0.911, 95%CI:0.54–2.00	
*AGTR1*or *FHIT* (High/Low)	Low, HR = 2.18, *P* = 0.069, 95%CI:0.94–5.05	

*High* Highly methylated cases, *Low* Lowly methylated cases, *N.S.* Not significant

### Association of p16 and RB expression with HPV infection status

Available immunohistochemical staining (IHC) data of p16 and RB were 89/94 (94.7%) and 54/94 cases (57.4%) respectively. The results of IHC were categorized into four groups. 1: Staining over 50% cells, 2: Staining in 20–50% cells, 3: Staining in 1–20% cells, and 4: Negative. Details of staining scores and the number of samples in each category are available in Supplementary Table S4a. We categorized ‘groups 1–2’ as IHC positive and ‘groups 3–4’ as IHC negative for easy tabulation. Forty-seven out of 89 cases (52.8%) were positive for p16 staining, and 25/54 (46.3%) cases were RB-positive staining (Supplementary Table S4b). Notably, 41/45 (91.1%) HPV-positive cases showed p16-positive staining, while 26/28 (92.9%) HPV-positive cases were RB-negative. As we defined HPV-positive cases in this study by PCR detection of HPV E6 and E7 mRNA detection method, a small number of HPV-positive cases might not indicate HPV-positive by IHC. Conversely, HPV-negative cases showed p16 negative staining (38/44, 86.4%) and RB positive (23/26, 88.5%) for most cases. As expected, HPV-positive cases were positively correlated with p16 expression, while HPV-negative cases were positively correlated with RB expression.

### Association of candidate gene promoter methylation with p16 or RB expression

To determine whether there is any correlation of p16 or RB expression with candidate gene methylation, the distribution of QMSP values among IHC positive and IHC groups was compared by the Mann–Whitney U test. Promoter methylation of *AGTR1*, *DAPK*, *FHIT* and *TIMP3* genes significantly associated with p16 positive cases (*P* = 0.045, *P* < 0.001, *P* < 0.001, *P* = 0.030). In contrast, *CSGALNACT2*, *DAPK* and *FHIT* promoter methylations were significantly associated with RB negative cases (*P* = 0.031, *P* < 0.001, *P* = 0.002, Supplementary Table S5). Table 4 summarizes the features of 8 candidate methylation markers. Previously reported 4 cancer-specific methylated genes(*DAPK*, *FHIT* and *TIMP3*) were HPV-positive associated OPSCC markers. Among the novel 4 genes tested in this study, *AGTR1* was strongly correlated with HPV-positive OPSCC characteristics.

**Table 4:** Characteristic summary of 8 methylated genes in OPSCCs

Gene	HPV positive OPSCC specificity	p16 expression (*n* = 89)	RB expression (*n* = 54)
***AGTR1***	**Highly methylated**	**High in p16 positive**	N.S
*CSGALNACT2*	N.S	N.S	**High in RB negative**
***DAPK***	**Highly methylated**	**High in p16 positive**	**High in RB negative**
***FHIT***	**Highly methylated**	**High in p16 positive**	**High in RB negative**
*GULP1*	N.S	N.S	N.S
*RASSF1A*	N.S	N.S	N.S
***TIMP3***	**Highly methylated**	**High in p16 positive**	N.S
*VGF*	**Highly methylated**	N.S	N.S

*High* Highly methylated, *Low* Lowly methylated, *N.S.* Not significant

### Candidate gene combinations specific to HPV-positive cancers

To improve the specificity of HPV-positive OPSCCs, we picked *AGTR1* and *FHIT* methylation markers based on AUC over 0.7 of ROC analysis results (Supplementary Figure S1). Either gene methylated cases were significantly associated with HPV-positive cancers with 92.0% sensitivity and 46.8% specificity, *P* < 0.001). Also, they had significantly better overall survival (*P* = 0.047, Supplementary Figure S2).

## Discussion

Viral infections cause human cancers, including liver, pharyngeal and cervical malignancies [25, 26]. Viruses disrupt the host cell biology and epigenetic process to promote replication and induce the deregulation of various gene promoter methylations [27]. As a result, aberrant DNA methylations are accumulated in non-cancerous or pre-cancerous tissues. It is a phenomenon, so-called the ‘epigenetic field for cancerization’ [28]. Hepatitis virus infection causes chronic hepatitis and induces epigenetic alterations associated with hepatoma risk [29]. Furthermore, the infection is proven to activate a natural killer cell-dependent innate immune response in mice and contribute to aberrant epigenetic accumulation [30].

Our findings solidified that promoter methylation of specific genes correlated with HPV viral infection. Here, we reported some novel genes frequently methylated in HPV-positive human OPSCCs. However, we do not know the precise mechanisms of how HPV infection induces these methylations and the biological effect of these methylations in carcinogenesis processes. The previous report suggests that the E6 and E7 oncoproteins of HPV increase DNA methyltransferase 1 (DNMT1) expression through p53 degradation (24), affecting the promoter methylation of relevant genes. Another group found that methylation arises as a host cell's defense mechanism to silence viral DNA [31]. Although we did not check the expression of each methylated gene in our cohort, TCGA data analysis proves that some of the analyzed gene methylations were associated with expression in OPSCCs.

Several DNA methylation markers have been identified in HPV-positive cervical cancers [32, 33]. Therefore, we examined eight candidate genes in OPSCCs in this study and summarized the result in Table 4. Based on optimal cut-off values, we found OPSCC-specific promoter methylation of *AGTR1*, *DAPK*, *FHIT*, *TIMP3* and *VGF*. Among the analyzed genes, promoter methylation of *RASSF1A* is less frequent in HPV-positive cases than HPV-negative cases, consistent with the previous report that promoter methylation of *RASSF1A* is inversely correlated with HPV infection in head and neck and cervical cancer [11, 34]. Although the cut-off values we used need to be validated in another similar cohort, our pilot study supports that some of our panel genes have the potential for future clinical use in different clinical contexts.

In HPV-positive HNSCCs, disruptive *p53* mutation is not common [35]. Among the available 28 HPV-positive OPSCCs, most cases showed RB down-regulation (26/28, 92.9%). There is an inverse relationship between p16 expression and RB downregulation (out of 26 RB down-regulated cases, 23 expressed p16, and only 3 cases negative for p16 expressions). This result means that p16 + /RB- phenotype is dominant in HPV-positive cases (23/28, 82.1%) following the previous reports [36–38]. Also, this subgroup is known as the high Ki-67 index subgroup in Basal-like carcinomas [39]. In our cohort, p16 + /RB- cases (*n* = 23) were compared with p16-/RB + cases (*n* = 24). Only negative smoking history (*P* = 0.002, Fisher’s exact test) was a significant factor of p16 + /RB- phenotype. Disease-specific survival of p16 + /RB- cases was significantly favorable than p16-/RB + cases (HR = 0.31, *P* = 0.008, 95%CI: 0.12–0.74). The promoter methylations of *AGTR1*, *CSGALNACT2*, *DAPK*, *FHIT* and *TIMP3* were significantly correlated with the p16 + /RB- phenotype (Table 4). However, there is no correlation between promoter methylation of individual genes and patients' survival. It may be interesting in future studies using a larger cohort to see whether promoter methylations of these genes have an additive effect with p16/RB status.

Among four novel methylation markers in OPSCC (*AGTR1*, *CSGALNACT2, GULP1* and *VGF*), *AGTR1* showed a significant correlation with cancer specificity, HPV infection specificity and p16 + /RB- phenotype (Table 4). *AGTR1* is a member of the angiotensin group of G-protein-coupled receptors that play a role in vasoconstriction, salt and water retention, cell proliferation and migration [40]. This gene is reportedly overexpressed in a subset of breast cancers [41, 42]. On the other hand, *AGTR1* methylation was significantly found in non-small cell lung cancer (NSCLC) [43] and colorectal cancer [15]. They showed that *AGTR1* methylation was found in 60% of NSCLC and 65% of colorectal cancers. Mitra et al. revealed that *AGTR1* was a target of oncogenic *miR-155*, and was silenced via *miR-155* up-regulation in head and neck cancer [44]. They also speculated that *AGTR1* expression was influenced by promoter hypermethylation.

A panel of OPSCC-specific and HPV-specific methylation markers can be available for early oropharyngeal cancer detection. One of the advantages of methylation marker detection is its high sensitivity and rapidity. We previously reported a rapid gene methylation detection procedure for head and neck cancers [45]. It takes less than three hours from sampling to get the result. We also reported that a few markers combination produces a definitive decision of cancer cell existence [46]. Thus, unlike conventional HPV PCR or p16 IHC examination, methylation detection analysis with excellent markers for sampled tissues or salivas has the potential to make a rapid and definitive diagnosis of malignancies originated from the HPV-infected field of cancerization. Furthermore, it may enable recurrent non-invasive examinations for high-risk patients or monitor postoperative advanced cancer patients.

There are some limitations of our study. First, since most cases were symptomatic and advanced and the samples came from surgically resected cases, the results may partially reflect the characteristics of advanced cancers. Second, an optimal cut-off of methylation level may be the cohort-specific value, which needs to be optimized by multiple data set testing. Third, we used a relatively small number of the single institute sample set, and data should be ascertained by more extensive multicenter cohort data. Finally, biological studies must be performed in contrast with HPV infection and promoter methylation.

In summary, we have detected a panel of novel HPV-positive and OPSCC-specific methylation markers. Especially, the combination of *ATGR1* methylation and *FHIT* methylation could be a sensitive biomarker of HPV-positive OPSCCs, which has a characteristic of the p16 positive and RB negative phenotypes.

## Supplementary Information

**Additional file 1: Supplementary Figure S1.** ROC Curves for determining optimal cut-off values between HPV-positive OPSCCs (*n*=50) and other samples including HPV-negative OPSCCs (*n*=44) and control samples (*n*=33). **Supplementary Figure S2.** Disease-specific survival curves of 94 OPSCCs depending on various clinicohistological factors, including HPV status.

**Additional file 2: Supplementary Table S1.** Candidate promoter methylated genes. **Supplementary Table S2.** Primers and probes for QMSP. **Supplementary Table S3.** Association of methylation markers with clinicopathological data (*n*=94). **Supplementary Table S4.** Classification of cases based on p16 and RB Staining. **Supplementary Table S5.** Correlation of QMSP values with p16 and RB expression (Mann-Whitney U test).

## Data Availability

The datasets generated and analyzed during the current study are not publicly available due to the ethical restrictions on surgery data and histological information. Still, they are available from the corresponding author upon reasonable request.

## Abbreviations

OPSCCs: HPV-positive oropharyngeal squamous cell carcinomas
QMSP: Quantitative-methylation specific PCR
AUC: Area under the curve
HPV: Human papillomavirus
RB: Retinoblastoma
HNSCCs: Head and neck squamous cell carcinomas
IHC: Immunohistochemical staining
