# Supplementary material for: High-risk HPV infection-associated hypermethylated genes in oropharyngeal squamous cell carcinomas
Source: BMC Cancer. 2022 Nov 7;22:1146. doi: 10.1186/s12885-022-10227-w (PMC9641857; doi:10.1186/s12885-022-10227-w)
Supplement: Supplementary file 1 — Additional file 1: Supplementary Figure S1. ROC Curves for determining optimal cut-off values between HPV-positive OPSCCs (n=50) and other samples including HPV-negative OPSCCs (n=44) and control samples (n=33). Supplementary Figure S2. Disease-specific survival curves of 94 OPSCCs depending on various clinicohistological factors, including HPV status. [file 12885_2022_10227_MOESM1_ESM.pptx]

## Slide 1
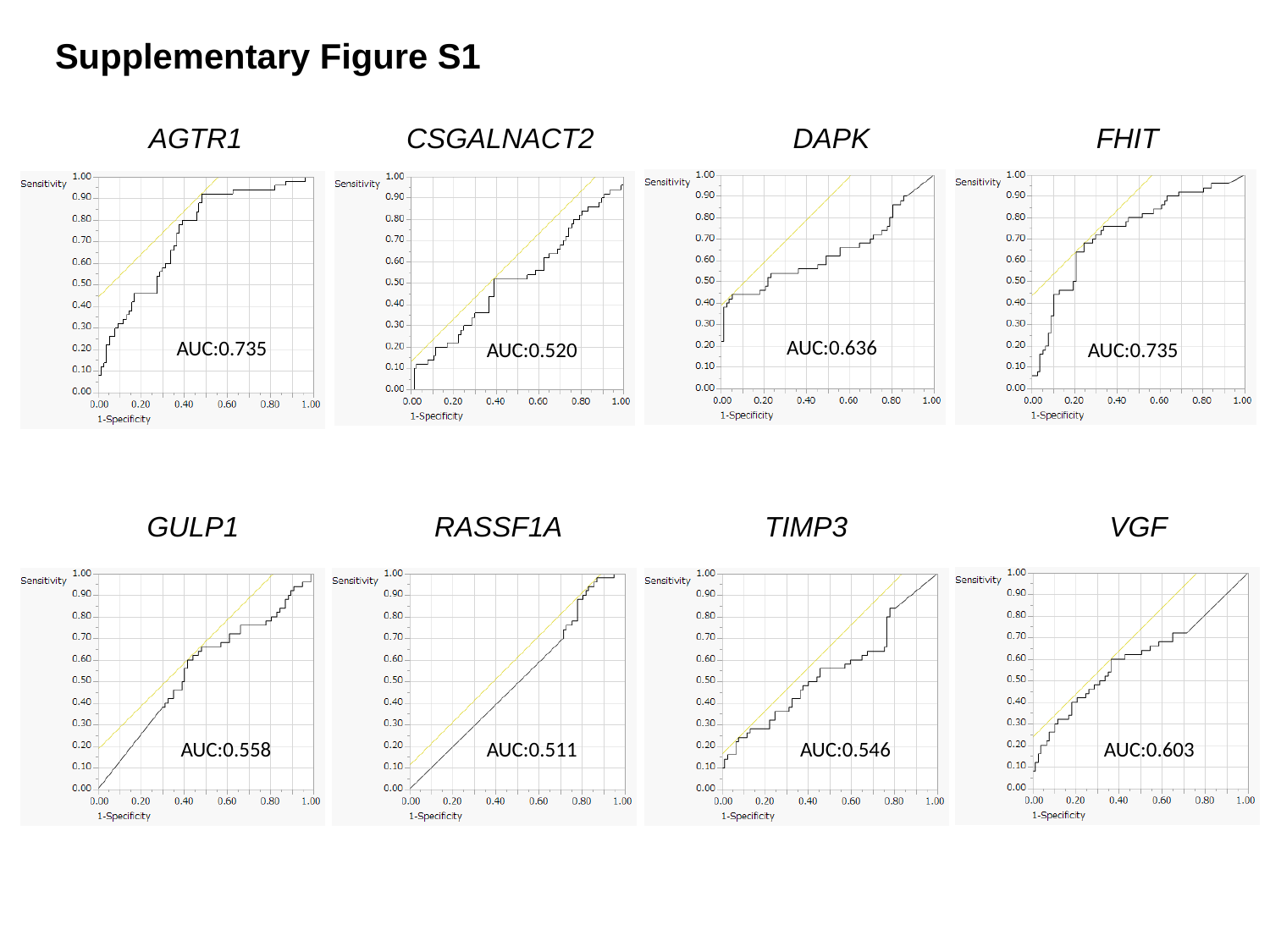

# Supplementary Figure S1
 AGTR1 CSGALNACT2 　DAPK FHIT
 GULP1 RASSF1A TIMP3 　 VGF
AUC:0.636
AUC:0.735
AUC:0.735
AUC:0.520
AUC:0.546
AUC:0.603
AUC:0.558
AUC:0.511

## Slide 2
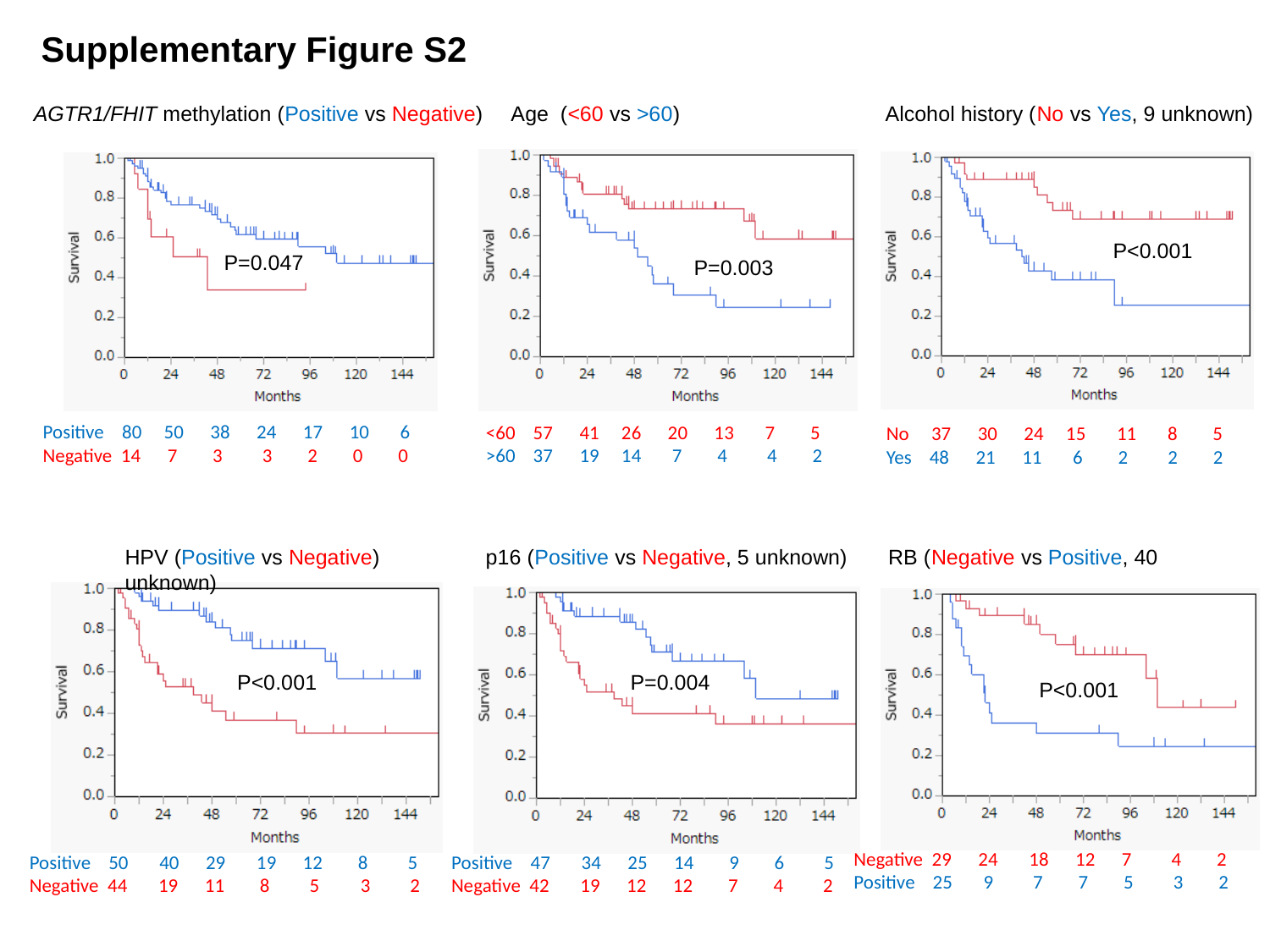

Supplementary Figure S2
AGTR1/FHIT methylation (Positive vs Negative) Age (<60 vs >60) Alcohol history (No vs Yes, 9 unknown)
P<0.001
P=0.047
P=0.003
Positive 80 50 38 24 17 10 6
Negative 14 7 3 3 2 0 0
<60 57 41 26 20 13 7 5
>60 37 19 14 7 4 4 2
No 37 30 24 15 11 8 5
Yes 48 21 11 6 2 2 2
HPV (Positive vs Negative) p16 (Positive vs Negative, 5 unknown) RB (Negative vs Positive, 40 unknown)
P<0.001
P=0.004
P<0.001
Negative 29 24 18 12 7 4 2
Positive 25 9 7 7 5 3 2
Positive 50 40 29 19 12 8 5
Negative 44 19 11 8 5 3 2
Positive 47 34 25 14 9 6 5
Negative 42 19 12 12 7 4 2
