# Supplementary material for: High-risk HPV infection-associated hypermethylated genes in oropharyngeal squamous cell carcinomas
Source: BMC Cancer. 2022 Nov 7;22:1146. doi: 10.1186/s12885-022-10227-w (PMC9641857; doi:10.1186/s12885-022-10227-w)
Supplement: Supplementary file 2 — Additional file 2: Supplementary Table S1. Candidate promoter methylated genes. Supplementary Table S2. Primers and probes for QMSP. Supplementary Table S3. Association of methylation markers with clinicopathological data (n=94). Supplementary Table S4. Classification of cases based on p16 and RB Staining. Supplementary Table S5. Correlation of QMSP values with p16 and RB expression (Mann-Whitney U test). [file 12885_2022_10227_MOESM2_ESM.docx]

**Supplementary Table S1** Candidate promoter methylated genes

| Methylated genes | Proposed functions | Chromosomal locus | Reported in human cancer | References |
| --- | --- | --- | --- | --- |
| *AGTR1* | Encodes a potent vasopressor hormone and a primary regulator of aldosterone secretion. | 3q24 | Colon | Carmona et al |
| *CSGALNACT2* | Encodes a protein that is involved in elongation during chondroitin sulfate synthesis. | 10q11.21 | N/A | N/A |
| *DAPK* | A positive mediator of gamma-interferon-induced programmed cell death. | 9q21.33 | Head and neck, lymphoma, Lung | Rosas et al, Rossi et al, Tang et al |
| *FHIT* | Encodes a diadenosine 5', 5'''-P1, P3-triphosphate hydrolase involved in purine metabolism. | 3p14.2 | Head and neck, Esophagus, Stomach, Colon. | Loyo et al, Tanaka et al, Schildhaus et al, Sinha et al |
| *GULP1* | An adapter protein that is necessary for the engulfment of apoptotic cells by phagocytes. | 2q32.3-q33 | Ovary, Bladder | Brait et al, Hayashi et al |
| *RASSF1A* | Encodes a protein similar to the RAS effector proteins, which interact with DNA repair protein XPA. It also inhibits the accumulation of cyclin D1 and induces cell cycle arrest. | 3p21.3 | Lung, Breast, Ovary, Bladder, Head and neck | Dammann et al, Dammann et al, Agathanggelou et al, Maruyama et al, Kwong et al |
| *TIMP3* | Encodes inhibitors of the matrix metalloproteinases, a group of peptidases involved in the extracellular matrix (ECM) degradation. | 22q12.3 | Head and neck, Esophagus, Liver, Stomach, Melanoma | Righini et al, Eads et al, Yu et al, Kang et al, van der Velden et al |
| *VGF* | Specifically expressed in a subpopulation of neuroendocrine cells, which is upregulated by nerve growth factor. | 7q22.1 | Breast, Ovary, Testis, Bladder | Ostrow et al, Brait et al, Hayashi et al |

**Supplementary Table S2** Primers and probes for QMSP

| Gene Symbol | F (5' - 3') | R (5' - 3') | Probe (6FAM 5' - 3'TAMRA) | Amplicon location | Annealing temperature |
| --- | --- | --- | --- | --- | --- |
| *ACTB* | TGGTGATGGAGGAGGTTTAGTAAGT | AACCAATAAAACCTACTCCTCCCTTAA | ACCACCACCCAACACACAATAACAAACACA | 390-522 | 60.0°C |
| *AGTR1* | TTTACGATTTTTCGTTAGGC | CTCCCTCTCGAAATATTAACG | TCAACGTTCACCTAATCC | -207 - -98 | 60.0°C |
| *CSGALNACT2* | TTAGTTGAGGGTCGTGGTCG | CGCACGTCTAACAAATACGCG | CGAACGCTACCTAAACCCCCGAA | -907 - -821 | 60.0°C |
| *DAPK* | GGA TAG TCG GAT CGA  GTT AAC GTC | CCC TCC CAA AC G CC G A | TTC GGT AA T TCG TAG CGG  TAG GGT TTG G | 4–102 | 60.0°C |
| *FHIT* | GGGCGCGGGTTTGGGTTTTTAC | GAAACAAAAACCCACCGCCCCG | AACGACGCCGACCCCACTAAACTCC | 192-293 | 60.0°C |
| *GULP1* | TGACGTTTGTTATGGTAGCG | TCCACGATTTCCCCACCG | CGAGGTCGGGGACGTAGCGG | 733-901 | 60.0°C |
| *RASSF1A* | GCG TTG AA G TCG GGG  TTC | CCC GTA CTT CGC TAA CTT  TAA AC G | ACA AAC GCG AAC CGA AC GAAA CCA | 45–119 | 60.0°C |
| *TIMP3* | GCGTCGGAGGTTAAGGTTGTT | CTCTCCAAAATTACCGTACGCG | AACTCGCTCGCCCGCCGA | 1051-1143 | 60.0°C |
| *VGF* | GGATAGCGTTCGTAGGCG | AAAAACCGAATTCCCCACCCCG | GCGCCCAAAAACGACGTAAACCTAAATAC | -502 - -418 | 60.0°C |

**Supplementary Table S3** Association of methylation markers with clinicopathological data (n=94)

|  | Age  (>60/<60) | Sex (Male/Female) | Race  (White/Other) | Smoking  (Yes/No) | Alcohol  (Yes/No) | Stage  (I-III/IV) | Grade  (1-2/3) |
| --- | --- | --- | --- | --- | --- | --- | --- |
| *AGTR1* | **0.014**  **(>60 > <60)** | N.S. | N.S. | N.S. | N.S. | N.S. | N.S. |
| *CSGALNACT2* | N.S. | N.S. | **0.007**  **(White>Other)** | N.S. | N.S. | N.S. | N.S. |
| *DAPK* | N.S. | N.S. | N.S. | N.S. | **0.017**  **(No>Yes)** | N.S. | N.S. |
| *FHIT* | N.S. | N.S. | **0.027**  **(White>Other)** | N.S. | N.S. | N.S. | N.S. |
| *GULP1* | N.S. | N.S. | **0.049**  **(Other>White)** | N.S. | N.S. | N.S. | N.S. |
| *RASSF1A* | **0.042**  **(>60 > <60)** | N.S. | N.S. | N.S. | N.S. | N.S. | N.S. |
| *TIMP3* | N.S. | N.S. | N.S. | N.S. | N.S. | N.S. | N.S. |
| *VGF* | N.S. | N.S. | N.S. | N.S. | N.S. | N.S. | **0.002**  **(1-2>3)** |

P values were analyzed by Fisher's exact test, two-tailed. N.S.: not significant

**Supplementary Table S4** Classification of cases based on p16 and RB Staining

1. Raw data

|  |  | p16 |  |  | RB |  |
| --- | --- | --- | --- | --- | --- | --- |
|  | Total | HPV positive | HPV negative | Total | HPV positive | HPV negative |
| 1: Strong over 50% | 40 | 37 | 3 | 13 | 0 | 13 |
| 2: Stained in 20-50% | 7 | 4 | 3 | 12 | 2 | 10 |
| 3: Weak | 1 | 1 | 0 | 17 | 15 | 2 |
| 4: Negative | 41 | 3 | 38 | 12 | 11 | 1 |
| Total | 89 | 45 | 44 | 54 | 28 | 26 |

1. Modified data (positive or negative)

|  |  | p16 |  |  | RB |  |
| --- | --- | --- | --- | --- | --- | --- |
|  | Total | HPV positive | HPV negative | Total | HPV positive | HPV negative |
| Positive (1-2) | 47 | 41 | 6 | 25 | 2 | 23 |
| Negative (3-4) | 42 | 4 | 38 | 29 | 26 | 3 |
| Total | 89 | 45 | 44 | 54 | 28 | 26 |

**Supplementary Table S5** Correlation of QMSP values with p16 and RB expression (Mann-Whitney U test)

| Gene | p16 IHC positive versus negative | | RB IHC positive versus negative | |
| --- | --- | --- | --- | --- |
| *AGTR1* | Highly methylated in p16 positive | **P=0.045** | N.S. | P=0.229 |
| *CSGALNACT2* | N.S. | P=0.084 | **Highly methylated in RB negative** | **P=0.031** |
| *DAPK* | Highly methylated in p16 positive | **P<0.001** | Highly methylated in RB negative | **P<0.001** |
| *FHIT* | Highly methylated in p16 positive | **P<0.001** | Highly methylated in RB negative | **P=0.002** |
| *GULP1* | N.S. | P=0.674 | N.S. | P=0.565 |
| *RASSF1A* | N.S. | P=0.060 | N.S. | P=0.229 |
| *TIMP3* | Highly methylated in p16 positive | **P=0.030** | N.S. | P=0.200 |
| *VGF* | N.S. | P=0.139 | N.S. | P=0.266 |

N.S.: not significant
